# Symptomatic and Asymptomatic *Campylobacter* Infections and Child Growth in South Asia: Analyzing Data from the Global Enteric Multicenter Study

**DOI:** 10.4269/ajtmh.22-0347

**Published:** 2023-05-01

**Authors:** Md. Iqbal Hossain, Sabiha Nasrin, Rina Das, Parag Palit, Al-Afroza Sultana, Rukaeya Amin Sobi, Soroar Hossain Khan, Sampa Dash, Mohammod Jobayer Chisti, Tahmeed Ahmed, Abu Syed Golam Faruque

**Affiliations:** ^1^ Nutrition and Clinical Services Division, International Center for Diarrheal Disease Research, Bangladesh, Dhaka, Bangladesh;; ^2^ James P. Grant School of Public Health, BRAC University, Dhaka, Bangladesh;; ^3^ Department of Biostatistics and Epidemiology, School of Public Health and Health Sciences, University of Massachusetts, Amherst, Massachusetts;; ^4^ Gangarosa Department of Environmental Health Sciences, Rollins School of Public Health, Emory University, Atlanta, Georgia;; ^5^ University of Virginia School of Medicine, Charlottesville, Virginia;; ^6^ Department of Global Health, University of Washington, Seattle, Washington

## Abstract

*Campylobacter* is a major cause of food-borne gastrointestinal illnesses worldwide, predominantly affecting children under 5 years of age. This study examined potential associations of symptomatic (with diarrhea) and asymptomatic (without diarrhea) *Campylobacter* infections with child growth among children under 5 years of age in South Asia. The Global Enteric Multicenter Study was conducted from 2007 to 2011 with a case-control design. Children were followed for 60 days after enrollment. Stool culture was performed to isolate *Campylobacter* spp. Among the 22,567 enrolled children, 9,439 were symptomatic, with 786 (8.28%) testing positive for *Campylobacter*. Conversely, 13,128 asymptomatic healthy controls were included, with 1,057 (8.05%) testing positive for *Campylobacter*. Growth faltering was observed in the symptomatic group, particularly among children aged 0–11 months (−0.19 height-for-age *z* score [HAZ]; 95% CI: −0.36, −0.03; *P* = 0.018) and 24–59 months (−0.16 HAZ; 95% CI: −0.28, −0.04; *P* = 0.010). However, in the asymptomatic group, growth faltering was observed only in the 24- to 59-month age group, in terms of HAZ (−0.15 HAZ; 95% CI: −0.24, −0.05; *P* = 0.002) and weight-for-height *z* score (−0.16; 95% CI: −0.26, −0.06; *P* = 0.001). These findings underscore the importance of immediate and enhanced introduction of preventive modalities to reduce the burden of *Campylobacter* infections and reduce their long-term sequelae.

## INTRODUCTION

*Campylobacter* is widely regarded as a leading causative agent for bacterial food-borne gastrointestinal illness worldwide.^1^^–^^3^ This enteric pathogen is significantly associated with moderate-to-severe diarrhea (MSD) among children aged 0–11 months in Bangladesh and Pakistan; and 24- to 59-month-old children in India and Pakistan.^2^ *Campylobacter* enteritis is an acute, self-limiting infection, and clinical manifestation ranges from watery diarrhea to dysenteric illnesses.^4^^,^^5^ This enteric infection damages gut mucosa, disrupts widespread gut commensal flora, and may even cause prolongation of the diarrheal episode.^6^ Long-term health sequelae include near-fatal Guillain–Barré syndrome, reactive arthritis, or Reiter’s syndrome.^7^ Postinfectious irritable bowel syndrome and inflammatory bowel disease are also linked with *Campylobacter* infection.^8^

Several studies have reported the isolation of *Campylobacter* as asymptomatic infections, which are common in older children.^9^^–^^11^ A study conducted in Peru reported that younger age, a recent history of diarrheal illness, and lower maternal education were linked to both asymptomatic and symptomatic *Campylobacter* infections. Some environmental factors in low- and middle-income countries (LMICs) pose a high risk of *Campylobacter* infections in humans, including poor sanitation, close contact with animals, and consumption of poultry meats and other poultry products.^12^^–^^14^ The isolation of this pathogen in nondiarrheal children could be explained by poor sanitation and early contact with animals.^13^ Moreover, excretion of *Campylobacter* by diarrheal infants has been reported in 75% of cases less than 7 days after diarrhea onset. However, such excretion may be prolonged, as high as 15 days and more among infants with both symptomatic infections (18%) and comparable asymptomatic colonization (11%) after diarrhea onset.^15^ Recent reports from the Etiology, Risk Factors, and Interactions of Enteric Infections and Malnutrition and the Consequences for Child Health and Development Project (MAL-ED) study have shown that the burden of asymptomatic infection by *Campylobacter* is associated with increased enteric inflammation.^16^ Previously, Chen et al. hypothesized that infections by certain species of the *Campylobacter* genus cause interruption of the gut mucosal barrier by targeting tight junctions and inducing a proinflammatory response in colonic epithelial cells, thereby leading to gastroenteritis.^17^ Recent studies have indicated that *Campylobacter* can use sufficient nutrients from the gut luminal environment for survival and growth.^18^ Asymptomatic malnourished children are more likely to carry *Campylobacter* in developing countries.^12^ This has led to the hypothesis that, it is an opportunistic infection in this setting, maybe linked to immunosuppression caused by malnutrition.^12^^,^^19^ A study of Peruvian Amazonian children observed reduced weight gain and marginally compromised linear growth over a 3- and 9-month period, respectively, in cases of both symptomatic and asymptomatic *Campylobacter* infections. Such observations were more pronounced in the case of more severe *Campylobacter* infections.^12^ Another study conducted in eight LMICs found *Campylobacter jejuni/coli* infections were associated with poor growth performance.^16^

However, information regarding the impact of *Campylobacter* infections on childhood growth in LMICs is lacking, especially in South Asia, where *Campylobacter* infection is a leading cause of MSD (moderate-to-severe diarrhea) episodes.^1^^,^^2^ In this study, our objectives were to evaluate potential associations of symptomatic and asymptomatic *Campylobacter* infections with child growth in three distinct countries in South Asia.

### Ethical considerations.

Study protocol approval was conferred by the institutional review board of the University of Maryland as well as by the research review committee and ethical review committee of the respective governmental or collaborating local institutions from each study site (Centro de Investigaçao em Saude da Manhiça, Manhiça, Mozambique; Medical Research Council, Basse, Gambia; CDC/Kenya Medical Research Institute Research Station, Kisumu, Kenya; Center pour le Développement des Vaccins du Mali, Bamako, Mali; National Institute of Cholera and Enteric Diseases, Kolkata, West Bengal, India; International Center for Diarrheal Disease Research, Bangladesh, Mirzapur, Bangladesh; Aga Khan University, Karachi, Pakistan). Before enrollment in the study, mothers/caregivers of eligible under-5 children were verbally informed about study objectives as well as the protocol; only those who gave written consent voluntarily were enrolled after stool specimens were provided. Subsequently, the mothers/caregivers were interviewed.

## MATERIALS AND METHODS

### Study sites.

The Global Enteric Multicenter Study (GEMS) was a 3-year prospective, age-stratified, matched case-control study of MSD (moderate-to-severe diarrhea) among children aged 0–59 months who sought care at sentinel hospitals and health centers in seven study sites in sub-Saharan Africa and South Asia.^21^ Children from the South Asian countries of the GEMS—Mirzapur, a rural community in Bangladesh; Kolkata, an urban site in India; and Karachi (Bin Qasim Town), a periurban site in Pakistan—were included in our analysis.

### Study design and participants.

The study was conducted from December 2007 to February 2011, and a case-control design was followed.^20^ Under-5 children from the demographic surveillance system catchment area presenting with MSD to the sentinel health center within 7 days of an acute illness onset were considered cases. Age-, sex-, and community-matched healthy controls were randomly selected from the community. Children with MSD and the healthy controls were enrolled and followed up after 60 days.^20^ Follow-up visits took place at the household of the participants. Nutritional assessments were performed at the time of enrollment (after hydration) as well as during follow-up visits.

### Specimen collection and laboratory procedure.

A fresh whole-stool sample (minimum 3 g) was collected from each enrolled child (both cases and controls). All stool samples were immediately shifted to the respective laboratory from each site following standard procedures of cold chain maintenance.^22^^–^^24^ Bacterial pathogens (*Salmonella, Shigella, Campylobacter* spp., *Aeromonas* spp., *Vibrio cholerae*, and *Escherichia coli* [enterotoxigenic (ETEC), enteropathogenic, and enteroaggregative (EAEC)]), viruses (rotavirus, norovirus, sapovirus, astrovirus, and adenovirus), and protozoa (*Entamoeba histolytica*, *Giardia intestinalis*, and *Cryptosporidium* spp.) were detected following standard laboratory methods as described elsewhere.^25^

In the GEMS, stool samples were delivered to the laboratory in cooler boxes after collection. A separate fecal aliquot was placed in two tubes, one containing Cary–Blair media^26^ and the other containing buffered glycerol saline (BGS),^27^ either at the time of collection or after the samples were received in the laboratory. When a fecal specimen could not be collected, a rectal swab was obtained during enrollment and promptly placed into the tubes containing Cary–Blair media and BGS. The study staff critically monitored the time between sample collection and transport to the laboratory, whereby the interval between stool collection and transport medium inoculation did not exceed 6 hours and the duration between placing the specimen in transport media and accession did not exceed 18 hours. For the following examinations, separate aliquots of the stool samples were made and stored in a −80°C freezer before further analysis.

### Fecal microbiology and *Campylobacter* spp. isolation.

The GEMS protocol incorporated traditional bacterial culture, largely to allow central laboratories to independently validate the growth of the involved pathogens and characterize them further for virulence, serologic, and antibiotic resistance features as described elsewhere.^25^ Swabs were plated onto Campy blood agar plates (Campy-BAPs) from the Cary–Blair tube. After observing the Campy-BAPs for growth on day 3 at 42°C, plates were further incubated. Suspicious colonies were chosen after incubation and subjected to a series of simple biochemical tests that could be easily carried out in resource-limited settings.^25^

### Data collection.

The passing of three abnormally loose or watery stools per 24 hours was defined as diarrhea.^20^ Many of the factors, such as vomiting (at least three times per day), fever (at least 38°C), and the presence of obvious blood in the stools, were examined.^20^ Both exclusively and partially breastfed children were referred to as “breastfed.” The information about the enrolled child’s family (defined as a group of people sharing a common cooking fire), included the mother as the major caregiver, maternal education (illiterate or literate), household size (including the number of children under the age of 5), building materials of the household (most common floor material: earth, sand, dung, and other), handwashing practices (before nursing or preparing baby food; after handling animals and cleaning a child), access to the main source of drinking water (tube well water and nontube well water), use of handwashing materials (water with soap or without soap), water treatment (water treatment method of drinking water available or not), improved toilet facilities (toilet facility for disposal of human fecal waste available or not), and presence of domestic animals or pets on the premises (sheep, goat, chicken, cow, dog, and cat), all of which were categorized as the explanatory variables in this analysis.^21^ To analyze possible factors linked with infection status (the presence of *Campylobacter* spp.), households were classified into socioeconomic status quintiles based on wealth index quintiles (poorest, lower middle, middle, upper middle, and richest).^20^ Global Enteric Multicenter Study field staff visited each enrolled child’s household approximately 60 days after enrollment, and detailed information on the morbidity of the participant was recorded as published elsewhere.^20^

### Anthropometry.

For each participant, height, weight, and mid-upper arm circumference (MUAC) were measured at enrollment and the 60-day household follow-up visit; details of the measuring methods have been described elsewhere.^20^ Using a digital scale calibrated every day (model 314, Tanita Corporation of America, Arlington Heights, IL), weight (to the nearest 0.1 kg) was measured following the standard guidelines for measurement. In the recumbent position, the length of children aged 0–23 months or those who were older but unable to stand unassisted were measured using a board with a fixed head and sliding foot piece (to the nearest 0.1 cm) (Shorr Productions, Olney, MD). For children aged 2 years and older, the same apparatus was used to measure standing height. To calculate the MUAC to the nearest 0.1 cm, a 25-cm paper single-slotted insertion tape was used (Shorr Productions).^28^ Length/height and MUAC were measured three times each and the average was estimated.^29^ Using WHO Child Growth Standards as the reference population, the height/length-for-age, weight-for-age, and weight-for-height/length *z* scores (HAZ, WAZ, and WHZ) were measured using a WHO SAS macro. After assessing the nutritional status of the children, *z* scores were calculated and categorized as underweight (WAZ < −2), stunted (HAZ < −2), or wasted (WHZ < −2) following the WHO anthropometry guidelines.^30^^,^^31^

### Data analysis.

We reported the child- and household-level characteristics by using mean and SD for continuous variables and frequency as a percentage for categorical variables to summarize the data. A paired *t* test was used to test the statistical significance of an observed difference between baseline and endline (follow-up measurement) *z* scores among these study children. To assess the association between the presence of *Campylobacter* spp. in stool among the symptomatic and asymptomatic children at baseline and the difference in the child’s HAZ, WAZ, and WHZ in the subsequent 60 days, we used a generalized linear model where the explanatory variable was the presence of *Campylobacter* in stool and the outcome variables were HAZ, WAZ, and WHZ. The repeated measure was used as the time variable in STATA (Stata Corporation, College Station, TX). We analyzed the difference in the *z* score from baseline to endline (60 days) by comparing the two measures over time. The explanatory variable (the presence of *Campylobacter* spp.) was used individually in the generalized linear model (simple linear regression) at first to investigate its unadjusted impact on the outcome variable (WAZ, HAZ, and WHZ). All the factors, namely age, sex, diarrhea at enrollment, breastfeeding status, mother’s education, number of under-5 children in the household, handwashing before nursing a child and after cleaning a child, handwashing material, main source of drinking water, wealth index, available toilet facility, co-pathogens (ETEC, *Shigella*, *Aeromonas*, and rotavirus), comorbidity (malaria, typhoid, pneumonia, diarrhea, and dysentery), time (because it was repeated measured data, the anthropometry was taken in two time points: on enrollment [0] and at day ∼60 follow-up [1]), and study site (country), suggesting the associations with the outcome as indicated in the literature were chosen for multivariable modeling (multiple logistic regression). The variance inflation factor (VIF) was calculated to detect multicollinearity, and no variable with a VIF value greater than 5 was identified in the final model. We estimated the coefficient and its 95% CI to describe the precision of the point estimate. During the analysis, a *P* value of < 0.05 was considered statistically significant. STATA 15.0 IC (Stata Corp LLC, College Station, TX) was used to analyze the entire dataset.

## RESULTS

**Figure 1.**
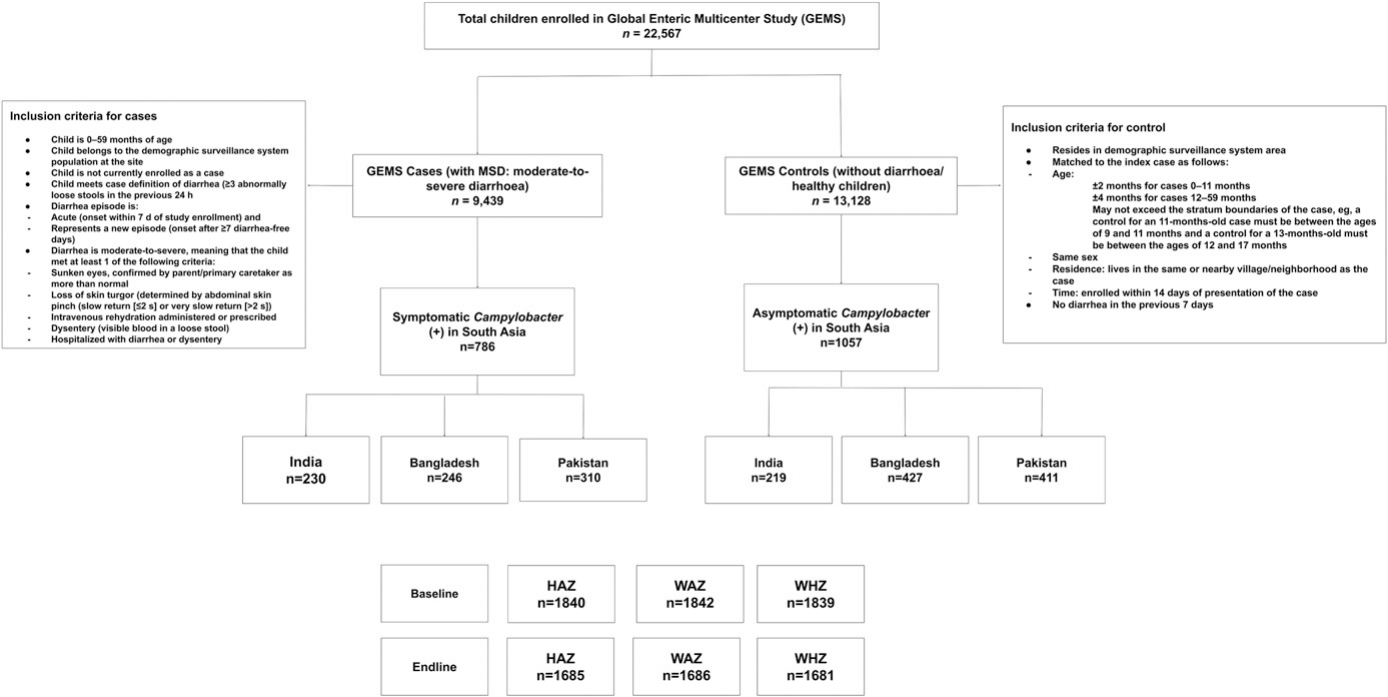
Study profile of children aged 0–59 months. HAZ = height-for-age *z* score; MSD = moderate-to-severe diarrhea; WAZ = weight-for-age *z* score; WHZ = weight-for-height *z* score.

**Figure 2.**
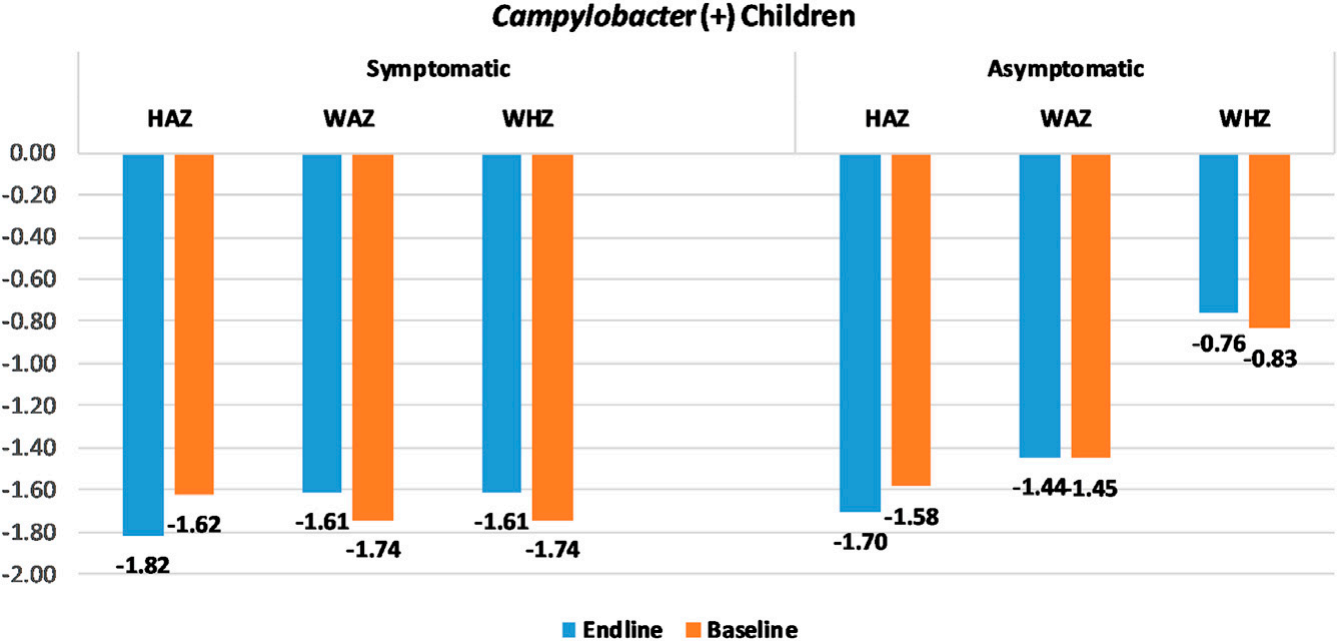
Mean baseline and endline height-for-age *z* score (HAZ), weight-for-age *z* score (WAZ), and weight-for-height *z* score (WHZ) among the fecal *Campylobacter* (+) symptomatic and asymptomatic children.

There were 9,439 cases (symptomatic children) and 13,128 healthy controls out of a total of 22,567 children in this study. Among the cases, 786 (8.28%) were positive for *Campylobacter* spp. (symptomatic *Campylobacter* spp. infection), whereas in the controls, 1,057 (8.05%) were positive for *Campylobacter* spp. (asymptomatic *Campylobacter* spp. infection) (Figure 1). In India, Bangladesh, and Pakistan, there were 230, 246, and 310 cases, respectively, and in the control group, there were 241, 427, and 411 individuals, respectively. There were 1,840 baseline data for HAZ, 1,842 for WAZ, and 1,839 for WHZ that could be used for analysis. However, we had 1,685, 1,686, and 1,681 data at 60 days of follow-up for HAZ, WAZ, and WHZ, respectively (Figure 1), because the height and weight of all the study participants could not be measured during baseline (the child’s parents were unwilling to provide the data) and endline during follow-up (dropout). Figure 2 shows the baseline and endline difference of HAZ, WAZ, and WHZ.

### Symptomatic *Campylobacter* (+) children.

Those children who were *Campylobacter* positive with diarrhea were enrolled as cases in the GEMS upon fulfilling the selection criteria as follows: they were between the ages of 0 and 59 months; belonged to the population covered by the demographic surveillance system at the study site; were not currently enrolled as a case; and met the case definition of diarrhea (≥ 3 abnormally loose stools in the previous 24 hours). The diarrhea episode also had to be acute (with onset within 7 days of study enrollment) and referred to as a new episode (onset after at least 7 diarrhea-free days); in addition, the child’s diarrhea had to be moderate to severe, as determined by at least one of the following criteria: sunken eyes as confirmed by the parent/primary caretaker as more than normal, loss of skin turgor as determined by an abdominal skin pinch (with slow return [≤ 2 seconds] or very slow return [> 2 seconds]), intravenous rehydration administered or prescribed, dysentery (visible blood in a loose stool), or hospitalization with diarrhea or dysentery.

### Asymptomatic *Campylobacter* (+) children.

Those children who were *Campylobacter* positive without diarrhea were enrolled as controls in the GEMS upon fulfillment of the following criteria: they resided in the demographic surveillance system area and were matched to the index case on the basis of age, sex, and time of enrollment within 14 days of presentation of the case. The age-matching criteria were ±2 months for cases aged 0–11 months and ±4 months for cases aged 12–59 months, with controls not exceeding the stratum boundaries of the case. In addition, controls must not have had diarrhea in the previous 7 days.

Table 1 illustrates that approximately 48% (*N* = 375/786) of symptomatic *Campylobacter* infection occurred in children aged 0–11 months, followed by 34% (*N* = 270/786) in children aged 12–23 months. In the asymptomatic group, 43% (*N* = 456/1,057) were in children aged 12–23 months followed by 34% (*N* = 361/1,067) in the 0- to 11-month age group. The symptomatic group had a higher percentage of underweight children (41%, *N* = 786), whereas the asymptomatic group had a higher percentage of children who were stunted (36%, *N* = 1,057). In both groups, the percentage of female sex and wealth index were similar.

**Table 1:** Sociodemographic characteristics and nutritional status of the study children

Sociodemographic characteristic	Symptomatic *Campylobacter* (+) children				Asymptomatic *Campylobacter* (+) children			
	India	Bangladesh	Pakistan	Total	India	Bangladesh	Pakistan	Total
	*N* = 230	*N* = 246	*N* = 310	*N* = 786	*N* = 219	*N* = 427	*N* = 411	*N* = 1,057
Age group (months), *n* (%)								
0–11	89 (38.70)	161 (65.45)	125 (40.32)	375 (47.71)	81 (36.99)	186 (43.56)	94 (22.87)	361 (34.15)
12–23	90 (39.13)	70 (28.46)	110 (35.48)	270 (34.35)	90 (41.10)	168 (39.34)	198 (48.18)	456 (43.14)
24–59	51 (22.17)	15 (6.10)	75 (24.19)	141 (17.94)	48 (21.92)	73 (17.10)	119 (28.95)	240 (22.71)
Sex (female), *n* (%)	90 (39.13)	104 (42.28)	140 (45.16)	334 (42.49)	91 (41.55)	176 (41.22)	187 (45.50)	454 (42.95)
Anthropometry (baseline), *n* (%)								
Stunted	83 (36.09)	48 (19.51)	163 (52.75)	294 (37.45)	63 (28.77)	109 (25.53)	204 (49.88)	367 (35.64)
Wasted	60 (26.09)	30 (12.20)	99 (32.14)	189 (24.11)	28 (12.79)	46 (10.77)	59 (14.43)	133 (12.61)
Underweight	93 (40.43)	52 (21.14)	177 (57.10)	322 (40.97)	55 (25.11)	100 (23.42)	173 (42.20)	328 (31.06)
Wealth index, *n* (%)								
Poorest	65 (28.26)	50 (20.33)	71 (22.90)	186 (23.66)	48 (21.92)	108 (25.29)	70 (17.03)	226 (21.38)
Lower middle	45 (19.57)	46 (18.70)	55 (17.74)	146 (18.58)	32 (14.61)	90 (21.08)	83 (20.19)	205 (19.39)
Middle	56 (24.35)	44 (17.89)	66 (21.29)	166 (21.12)	55 (25.11)	77 (18.03)	88 (21.41)	220 (20.81)
Upper middle	31 (13.48)	46 (18.70)	54 (17.42)	131 (16.67)	44 (20.09)	75 (17.56)	79 (19.22)	198 (18.73)
Richest	33 (14.35)	60 (24.39)	64 (20.65)	157 (19.97)	40 (18.26)	77 (18.03)	91 (22.14)	208 (19.68)

The top five copathogens identified with *Campylobacter* spp. infections were ETEC (24%), *Aeromonas* (23%), EAEC (19%), *Cryptosporidium* (19%), and *Giardia lamblia* (18%), as shown in Table 2. Except for *Cryptosporidium*, EAEC, and *Giardia*, there were significant differences in all copathogens in both groups.

**Table 2:** Copathogens isolated from stool samples of the *Campylobacter* (+) children

Pathogens isolated	Frequency, *n* (%)	*P* value
*Campylobacter* + *Aeromonas*	281 (22.39)	< 0.001
*Campylobacter* + EAEC	370 (18.81)	0.088
*Campylobacter* + *Cryptosporidium*	176 (18.6)	0.345
*Campylobacter* + *Shigella*	102 (11.04)	< 0.001
*Campylobacter* + ETEC	167 (24.03)	< 0.001
*Campylobacter* + *Giardia*	376 (18.25)	0.319
*Campylobacter* + rotavirus	148 (14.45)	0.007

EAEC = enteroaggregative *Escherichia coli*; ETEC = enterotoxigenic *Escherichia coli*. Results are from χ^2^ tests.

The outcomes of the multiple linear regression model are presented in Table 3. After adjusting for sex, breastfeeding status, maternal education, handwashing before nursing a child and after cleaning the child, handwashing material, main source of drinking water, available toilet facility, wealth index, co-pathogens (ETEC, *Shigella*, *Aeromonas*, and rotavirus), study site, and comorbidity (malaria, typhoid, pneumonia, diarrhea, and dysentery), significant growth faltering was observed in the symptomatic group (MSD children, who were enrolled as cases) among infants and young children aged 0–11 months (−0.19 HAZ difference in 60-day follow-up; 95% CI: −0.36, −0.03; *P* = 0.018) and 24–59 months (−0.16 HAZ difference; 95% CI: −0.28, −0.04; *P* = 0.010). However, in the asymptomatic group (healthy children without having diarrhea, enrolled as controls from the community), growth faltering was observed only in the 24- to 59-month age group (−0.15 HAZ difference; 95% CI: −0.24, −0.05; *P* = 0.002; −0.16 WHZ difference; 95% CI: −0.26, −0.06; *P* = 0.001) (Table 3).

**Table 3:** Association of symptomatic and asymptomatic *Campylobacter* infections with the difference in children’s HAZ, WAZ, and WHZ

Age	Symptomatic *Campylobacter* (+)				Asymptomatic *Campylobacter* (+)			
	Unadjusted		Adjusted*		Unadjusted		Adjusted*	
	Coef. (95% CI)	*P* value	Coef. (95% CI)	*P* value	Coef. (95% CI)	*P* value	Coef. (95% CI)	*P* value
0–11 months								
HAZ	−0.52 (−0.69, −0.36)	< 0.001	−0.19 (−0.36, −0.03)	**0.018**	−0.22 (0.33, 0.1)	< 0.001	−0.04 (−0.15, 0.07)	0.515
WAZ	−0.32 (−0.47, −0.17)	< 0.001	−0.12 (−0.27, 0.03)	0.114	−0.06 (0.16, 0.05)	0.286	0.06 (−0.05, 0.16)	0.302
WHZ	−0.04 (−0.19, 0.11)	0.593	−0.03 (−0.18, 0.13)	0.709	0.09 (0, 0.19)	0.061	0.10 (−0.003, 0.20)	0.058
12–23 months								
HAZ	−0.02 (−0.12, 0.09)	0.772	−0.02 (−0.12, 0.08)	0.696	0.02 (−0.08, 0.12)	0.757	0.04 (−0.06, 0.14)	0.395
WAZ	−0.02 (−0.14, 0.09)	0.669	−0.03 (−0.14, 0.08)	0.580	−0.04 (0.14, 0.07)	0.511	−0.002 (−0.11, 0.10)	0.969
WHZ	−0.05 (−0.17, 0.07)	0.400	−0.06 (−0.18, 0.06)	0.340	−0.13 (−0.24, 0.02)	0.024	−0.11 (−0.22, 0.003)	0.058
24–59 months								
HAZ	−0.32 (−0.45, −0.19)	< 0.001	−0.16 (−0.28, −0.04)	**0.010**	−0.18 (−0.27, 0.09)	< 0.001	−0.07 (−0.16, 0.02)	0.136
WAZ	−0.20 (−0.32, −0.08)	0.002	−0.03 (−0.16, 0.09)	0.580	−0.26 (−0.35, 0.16)	< 0.001	−0.15 (−0.24, −0.05)	**0.002**
WHZ	−0.04 (−0.17, 0.08)	0.486	0.07 (−0.06, 0.19)	0.294	−0.23 (−0.33, 0.14)	< 0.001	−0.16 (−0.26, −0.06)	**0.001**

Coef. = coefficient; HAZ = height-for-age *z* score; WAZ = weight-for-age *z* score; WHZ = weight-for-height *z* score. Results of multiple linear regression modeling (the independent variable is the presence or absence of *Campylobacter* and dependent variables—HAZ, WAZ, and WHZ) among the different age groups. Separate models were performed to see the association of *Campylobacter* infection with a child’s HAZ, WAZ, and WHZ for symptomatic and asymptomatic infections. Bolding indicates significant values.

* Adjusted for sex, breastfeeding status, mother’s education, handwashing before nursing a child and after cleaning a child, handwashing material, main source of drinking water, available toilet facility, wealth index, copathogens (enterotoxigenic *Escherichia coli*, *Shigella*, *Aeromonas*, and rotavirus), sites (country), and comorbidity (malaria, typhoid, pneumonia, diarrhea, and dysentery). *Campylobacter* was detected from the stool samples during enrollment. Anthropometric measurements were taken during enrollment and 60 days after enrollment (during the follow-up visit).

## DISCUSSION

The study findings supported our hypothesis that both symptomatic and asymptomatic infections by *Campylobacter* were associated with growth faltering in under-5 children in South Asia. Important findings concerning these children with symptomatic and asymptomatic *Campylobacter* infections include 1) *Campylobacter* infections were equally distributed among study children with MSD and children without diarrhea, 2) growth faltering was observed during a follow-up period of approximately 60 days, and 3) co-pathogens that were commonly detected with both symptomatic and asymptomatic *Campylobacter* infections were ETEC, *Aeromonas*, EAEC, *Cryptosporidium, G. lamblia*, and *Shigella*. We observed reduced growth with both symptomatic and asymptomatic *Campylobacter* infections in this cohort from the Indian subcontinent. However, after controlling for seasonality and potential confounders such as socioeconomic and demographic factors, one prospective multisite birth cohort study found an association between *Campylobacter* infection and poor linear growth in children under the age of 2 years, suggesting that *Campylobacter* may play a role in childhood malnutrition.^16^

*Campylobacter* is a bacterial cause of diarrhea that has been reported in infants and young children in both industrialized and developing countries.^32^^,^^33^ In this study, control children were diarrhea free for the previous 7 days at the time of enrollment in the study. Cases and controls were equally infected with *Campylobacter* spp. All these observations could be explained in several ways; these controls may have experienced symptomatic *Campylobacter* infections in the recent past. Despite recovery from the diarrheal episode, these control children continued to excrete *Campylobacter* asymptomatically for an extended period (more than 7 days after onset). However, we do not have any data because the study did not take any proper history in support of our statement. Like other enteric pathogens in circulation, *Campylobacter* may have heterogeneous circulation, and some study children may have had an infection with those strains that were nonpathogenic or less pathogenic, and despite similar infective doses they were not able to develop manifestations of clinical diarrhea-like pathogenic strains.

Experimental challenge studies in healthy adult volunteers have indicated that the presence of any less pathogenic or nonpathogenic microorganism as a single infection may cause the manifestation of a relatively milder diarrheal episode or no episode at all.^34^ One study reported that both the susceptibility of the particular hosts and bacterial variables have an impact on the development of campylobacteriosis.^35^ Numerous virulence genes involved in invasion, colonization of the intestinal mucosa, motility, adhesion, and toxin synthesis may be found in the *Campylobacter* genome. It was depicted that despite the cytopathic effects on epithelial cells, the capacity of bacteria to enter host cells is frequently not the primary mechanism leading to infection.^35^ Another study reported that there are numerous species of *Campylobacter* spp.^36^ However, *C. jejuni/coli* infection is predominantly linked to *Campylobacter* enteritis in humans. The pathogenicity of non-*jejuni/coli Campylobacter*-like *Campylobacter upsaliensis* and *Campylobacter helveticus* (isolated from dogs and cats) is unknown. In our study, some children may have had an infection with *Campylobacter* as the sole pathogen, and in the absence of pathogenicity or because of being less pathogenic any overt clinical illness was not manifested. Moreover, due to the failure to use molecules present on the surface of human intestinal cells as specific receptors, there were no disease manifestations other than asymptomatic infections. Studies have also revealed the role of functional barriers, the mucus layer, gut microbiota, antimicrobial peptides, and immune defense mechanisms in conferring protection against diarrheal episodes.^4^^,^^34^^,^^36^^–^^39^

Reduced growth and poor nutritional status among children due to the increased burden of diarrhea are well reported.^40^^–^^42^ Malnutrition in turn increases vulnerability to diarrhea and is often accompanied by malabsorption, small bowel overgrowth, increased intestinal permeability, enteropathy, gram-negative (enteric) bacteremia, and suboptimal immune response.^19^^,^^43^^–^^50^ *Campylobacter*-associated episodes are acute but self-limiting and more common among malnourished children.^43^^,^^51^^,^^52^ It has also been reported that asymptomatic *Campylobacter* infection was more common among malnourished children.^19^ Both symptomatic and asymptomatic infections were associated with the excretion of *Campylobacter* for a longer duration.^15^^,^^53^ The adverse effects on gut mucosa due to *Campylobacter* infections may even extend beyond the period of acute infection. Chen et al. reported that disrupted barrier function and increased cytokine secretion by intestinal epithelial cells attract neutrophils, macrophages, and lymphocytes to the gut inflammation site, causing intensification of host responses that may result in uncontrolled postinfectious intestinal complications including irritable bowel syndrome.^17^

Malnourished asymptomatic children were more often infected with *Campylobacter* than symptomatic children, and this is potentially caused by the presence of their host susceptibility factors and the absence of nonspecific host factors that can confront diarrhea pathogens.^50^^,^^54^ Well-nourished symptomatic children were infected with *Campylobacter* more frequently than asymptomatic well-nourished children, as *Campylobacter* has been revealed to be able to obtain sufficient nutrients from the host’s gut environment for survival and rapid growth in the intestinal lumen.^18^ A prospective cohort study of 442 children aged 0–72 months conducted in a semirural community in the Peruvian Amazon reported the nutritional consequences of *Campylobacter* infections. The study observed reduced weight gain due to asymptomatic and symptomatic *Campylobacter* infections over 3 months. However, symptomatic *Campylobacter* infections were marginally associated with reduced linear growth over 9 months. The study further revealed that relatively severe episodes were associated with reduced linear growth.^12^

The present study has several strengths. Data were collected from a relatively large number of children presenting with MSD and their concurrent controls and the study gathered a variety of information. The study focuses on health institutions from three distinct South Asian sites. The strengths of our study also include unbiased random sampling and high-quality laboratory performance. A notable feature of this study was the single follow-up household visit, roughly 60 days after enrollment, and consequently the observed association may be due to an acute effect. Separate analyses for *C. jejuni/coli*, a pathogenic variant of *Campylobacter* spp., were not possible; this was a limitation of our study.

## CONCLUSION

Both symptomatic and asymptomatic *Campylobacter* infections were associated with growth faltering in under-5 children in South Asia. High preventive public health priorities from different sectors of health policy are imperative to reduce the burden of childhood symptomatic and asymptomatic *Campylobacter* spp. infections, and thereby their nutritional consequences.

## Financial Disclosure

This work was supported, in whole or in part, by the Bill & Melinda Gates Foundation [INV-002050]. Under the grant conditions of the Foundation, a Creative Commons Attribution 4.0 Generic License has already been assigned to the Author Accepted Manuscript version that might arise from this submission.
